# Immunological dynamics in orthotopic compared with subcutaneous murine models of HPV-positive oropharyngeal cancer

**DOI:** 10.1242/dmm.052311

**Published:** 2025-12-05

**Authors:** Minzi Mao, Ke Qiu, Lan Feng, Yao Song, Yufang Rao, Shuo Li, Danni Cheng, Xiuli Shao, Chuanhuan Jiang, Shenglan You, Wei Xu, Geoffrey Liu, Jadwiga Jablonska, Stephan Lang, Shuaicheng Li, Fei Chen, Yu Zhao, Jianjun Ren

**Affiliations:** ^1^ Department of Oto-Rhino-Laryngology, West China Hospital, Sichuan University, Chengdu, 610041 Sichuan, China; ^2^ Research Core Facility of West China Hospital, Sichuan University, Chengdu, 610041 Sichuan, China; ^3^ Animal Imaging Core Facilities, West China Hospital, Sichuan University, Chengdu, 610041 Sichuan, China; ^4^ Department of Biostatistics, Princess Margaret Cancer Centre and Dalla Lana School of Public Health, M5G 2C1 Toronto, Ontario, Canada; ^5^ Department of Medicine, Division of Medical Oncology and Hematology, Princess Margaret Cancer Center, University Health Network, University of Toronto, M5G 2C1 Toronto, Canada; ^6^ Translational Oncology, Department of Otolaryngology, University Hospital Essen, University of Duisburg-Essen, 45147 Essen, Germany; ^7^ Department of Computer Science, City University of Hong Kong, 999077 Hong Kong, China

**Keywords:** Tumor immune microenvironment (TIME), Human papillomavirus (HPV)-positive head and neck squamous cell carcinoma (OPSCC), Murine models, Drug sensitivity

## Abstract

The necessity of reliable preclinical models for evaluating the efficacy of novel therapeutic strategies is imperative. Nevertheless, the degree to which tumor-bearing murine models represent the immunological characteristics of human papillomavirus (HPV)-positive oropharyngeal squamous cell carcinoma (OPSCC) has largely been unexplored. By utilizing single-cell RNA sequencing technology, our research elucidated that subcutaneous (SC) murine models more accurately reflect the early immunogenic phase of human HPV-positive OPSCC, marked by a stage-dependent increase in effector T-cell infiltration. By contrast, orthotopic (base of tongue, BOT) tumors exhibited a progressive decline of cytotoxic T cells and accumulation of myeloid-derived suppressive cells, paralleling the immune decrease observed in advanced, immune-excluded human tumors. Additionally, our drug responsiveness analysis indicated that early-stage BOT models more accurately replicate the response to PDCD1 blockade, whereas late-stage SC models more accurately mirror the response to CTLA4 blockade akin to human samples. Our findings provide pivotal insights into the suitability of murine models for the preclinical assessment of immunotherapies in HPV-positive OPSCC.

## Teaser

**Summary:** Our study reveals distinct immune dynamics in orthotopic and subcutaneous human papillomavirus (HPV)-positive oropharyngeal squamous cell carcinoma (OPSCC) models, offering insights for optimizing preclinical immunotherapy and refining cancer immunology research.

## INTRODUCTION

Head and neck squamous cell carcinoma (OPSCC) is the sixth most common cancer worldwide (890,000 new cases and 450,000 deaths annually), and the incidence of OPSCC continues to rise, primarily due to the increase in human papillomavirus (HPV) infection in younger individuals (Ferlay et al., 2019; Lechner et al., 2022).

The tumor immune microenvironment (TIME) is a crucial determinant of cancer progression and response to therapies, playing an important role in shaping the immune landscape and influencing tumor dynamics (Welters et al., 2020). The TIME in HPV-positive OPSCC comprises a complex interplay of various types of immune cell, including tumor-associated macrophages (TAMs), tumor-associated neutrophils (TANs) and regulatory T cells (Tregs), all of which contribute to an immunosuppressive environment (Ferris and Westra, 2023). Understanding how these cell populations interact and evolve during tumor progression is essential for developing effective immunotherapies, particularly immune checkpoint blockades (ICBs) that have shown promise for treating other malignancies. However, heterogeneity of the TIME can complicate the evaluation of therapeutic responses.

Murine models are valuable tools to investigate the TIME and to evaluate preclinical therapeutic interventions (Guerin et al., 2020). Although subcutaneous models have been widely used because of their ease of implantation and monitoring, orthotopic models are believed to better mimic the native tumor microenvironment and metastatic behavior (Chaves et al., 2023; Zhou et al., 2024). Despite their potential advantages, the extent to which these models recapitulate the immunological features of human HPV-positive OPSCC remains largely unknown.

In this study, we aimed to comprehensively characterize the TIME within orthotopic – in our research the base of tongue (hereafter referred to as BOT) – and subcutaneous (SC) murine models of HPV-positive OPSCC, and to compare them with immunological features in humans. By using single-cell RNA sequencing (scRNA-seq) of CD45-positive (CD45^+^) immune cells isolated from early- and late-stage tumors, we sought to delineate the functional profiles of various subsets of immune cells and identify fundamental immunological dynamics during tumor progression. We also examined the expression of inhibitory receptor genes within the T-cell subpopulations and assessed their sensitivity to canonical ICBs. Our findings may provide critical insights into the immune landscapes within these models and their relevance for preclinical drug sensitivity assessments, ultimately contributing to the advancement of therapeutic strategies for HPV-positive OPSCC.

## RESULTS

### scRNA-seq identifies dynamic immune cell heterogeneity during HPV-positive OPSCC progression

**Fig. 1.**
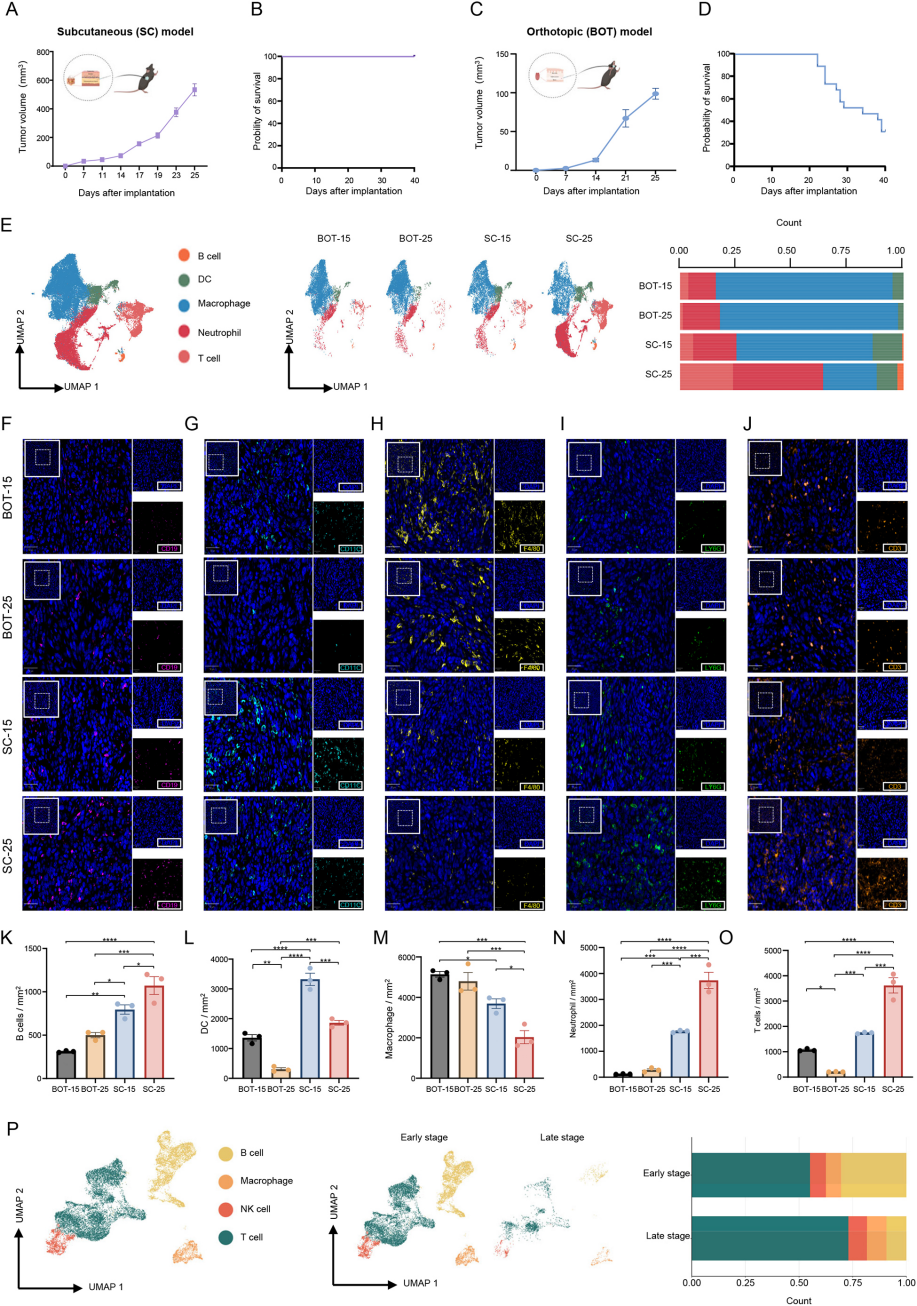
**scRNA-seq identifies dynamic immune cell heterogeneity during HPV-positive OPSCC progression.** (A) Tumor growth progression in a subcutaneous (SC) murine model (*n*=11/group). The schematic was created in BioRender.com. (B) Survival curves for the SC murine model (*n*=11/group). (C) Tumor growth progression in an orthotopic (BOT) murine model (*n*=9/group). Created in BioRender by Mao, M., 2025. https://BioRender.com/yif7a76. Republished with permission. (D) Survival curves for the BOT murine model (*n*=9/group). (E) Left: Uniform manifold approximation and projection (UMAP) plot (left) illustrating five main immune cell clusters identified in SC and BOT murine models, i.e. B cells (orange), dendritic cells (DCs, green), macrophages (blue), neutrophil (red) and T cells (coral). Middle: UMAP plots showing the distribution of single cells at early and late stages of both models. Right: bar chart showing the distribution of these five clusters at early (day 15) and late (day 25) stages of tumor development in SC and BOT models. (F-J) Representative immunofluorescence images of SC and or BOT tumors harvested at day 15 and day 25 post implantation. Sections stained for B cells (F), DCs (G), macrophages (H), neutrophils (I) and T cells (J). A panoramic view is shown in the top-left panel (scale bars: 100 μm), with a region of interest (ROI) boxed. This ROI is shown magnified (×5) in the bottom-right corner, which depicts the corresponding merged image. Scale bars for the magnification: 50 μm. (K-O) Quantification of tumor-associated density of B cells (K), DCs (L), macrophages (M), neutrophils (N) and T cells (O) per mm² tumor area for each group. Data are presented as the mean±s.e.m. (five fields/mouse; *n*=3 mice/group). Statistical significance was determined by two-way ANOVA with Tukey's post-hoc test: *P<0.05, **P<0.01, ***P<0.001 and ****P<0.0001. (P) Left: UMAP plot showing four main immune cell clusters identified in human HPV-positive OPSCC, i.e. B cells (yellow), macrophages (orange), natural killer (NK) cells (red) and T cells (dark green). Middle: UMAP plots showing the distribution of single cells at different clinical stages. Right: bar chart showing the distribution of these clusters across different clinical stages in human HPV-positive OPSCC.

To investigate the heterogeneity of immune cell populations and the functional consequence of this diversity in the TIME during HPV-positive OPSCC malignant progression, we used both BOT and SC murine models established by implantation of the most commonly used HPV-positive mEERL cell line derived from mouse oropharyngeal epithelium (Dorta-Estremera et al., 2018; Hanoteau et al., 2019; Smalley Rumfield et al., 2020). Specifically, our SC models were established by implanting tumor cells in the subcutaneous tissue on the right flank (Fig. 1A, Fig. S1A), enabling the rapid growth of tumors with an average size of ∼600 mm^3^ on day 25 following implantation (Fig. 1A). No fatalities were recorded at the end of the study period (Fig. 1B). BOT models, by contrast, were established by implanting tumor cells in the submucosal area at the base of the tongue (Fig. 1C, Fig. S1B), and showed slow tumor growth but high fatality rates due to obstruction of the respiratory tract (Fig. 1D).

We then characterized the dynamics of the immune-cell landscape during tumor progression by performing scRNA-seq on isolated CD45^+^ cells of five pooled tumor tissues obtained from early stage and late-stage (days 15 and 25, respectively) SC and BOT models. Following quality control, a total of 42,736 immune cells were retained for downstream analysis, i.e. 26,429 cells from SC tumors and 16,307 cells from BOT tumors. across both models, five main types of immune cell were identified by using canonical markers, i.e. 606 B cells, 3359 dendritic cells (DCs), 22,054 macrophages, 11,390 neutrophils and 5327T cells were detected (Fig. 1E, Fig. S1C, Table S1). Notably, SC and BOT models exhibited similar TIMEs, with the majority of intratumoral immune cells being myeloid cells, including macrophages and neutrophils. Additionally, increased neutrophil infiltration was found to be correlated with tumor progression in both models, which could be associated with poor prognosis and limited efficacy of immune therapeutics (Correia et al., 2021). Nevertheless, opposite trends of T-cell infiltration were identified in these two models, i.e. an increased proportion of T cells was observed during tumor progression in SC models, whereas T cells within the TIME of BOT models seemed to be eliminated during tumor progression, indicating worse outcomes because tumor-infiltrating T cells are the central players in recognizing and killing tumor cells (Quah et al., 2023; Prokhnevska et al., 2023; Yao et al., 2024). We also performed multiplex immunohistochemistry (mIHC) experiments on individual tumors (*n*=3 mice per group/stage) to provide quantitative spatial validation of these findings (Fig. 1F-O), which demonstrated similar progressive trends as scRNA-seq data.

To further assess the extent to which these models might recapitulate the immunological characteristics of human HPV-positive OPSCC, we performed scRNA-seq analysis of a cohort of human HPV-positive OPSCCs covering early- and late-stage samples (early stage: T1-T2, *n*=15; late stage: T3-T4, *n*=3). After quality control, a total of 17,380 immune cells was retained for downstream analysis. Four main types of immune cell were identified by using canonical markers, i.e. 4842 B cells, 1268 macrophages, 1312 natural killer (NK) cells and 9958 T cells were detected across all samples (Fig. 1P, Fig. S1D, Tables S2 and S9). Notably, we identified a lymphoid-dominant TIME in human samples, in which increased infiltration of T cells correlated with tumor progression, showing certain similarities with SC models.

Taken together and compared to the lymphoid-dominant TIME of human HPV-positive OPSCC, the SC and BOT models were characterized by more myeloid-dominant TIMEs. However, SC models showed a stage-dependent increase in T-cell infiltration, partially recapitulating the immunological features of human HPV-positive OPSCC.

### Distinct T-cell dynamics between murine models and human HPV-positive OPSCC during tumor progression

**Fig. 2.**
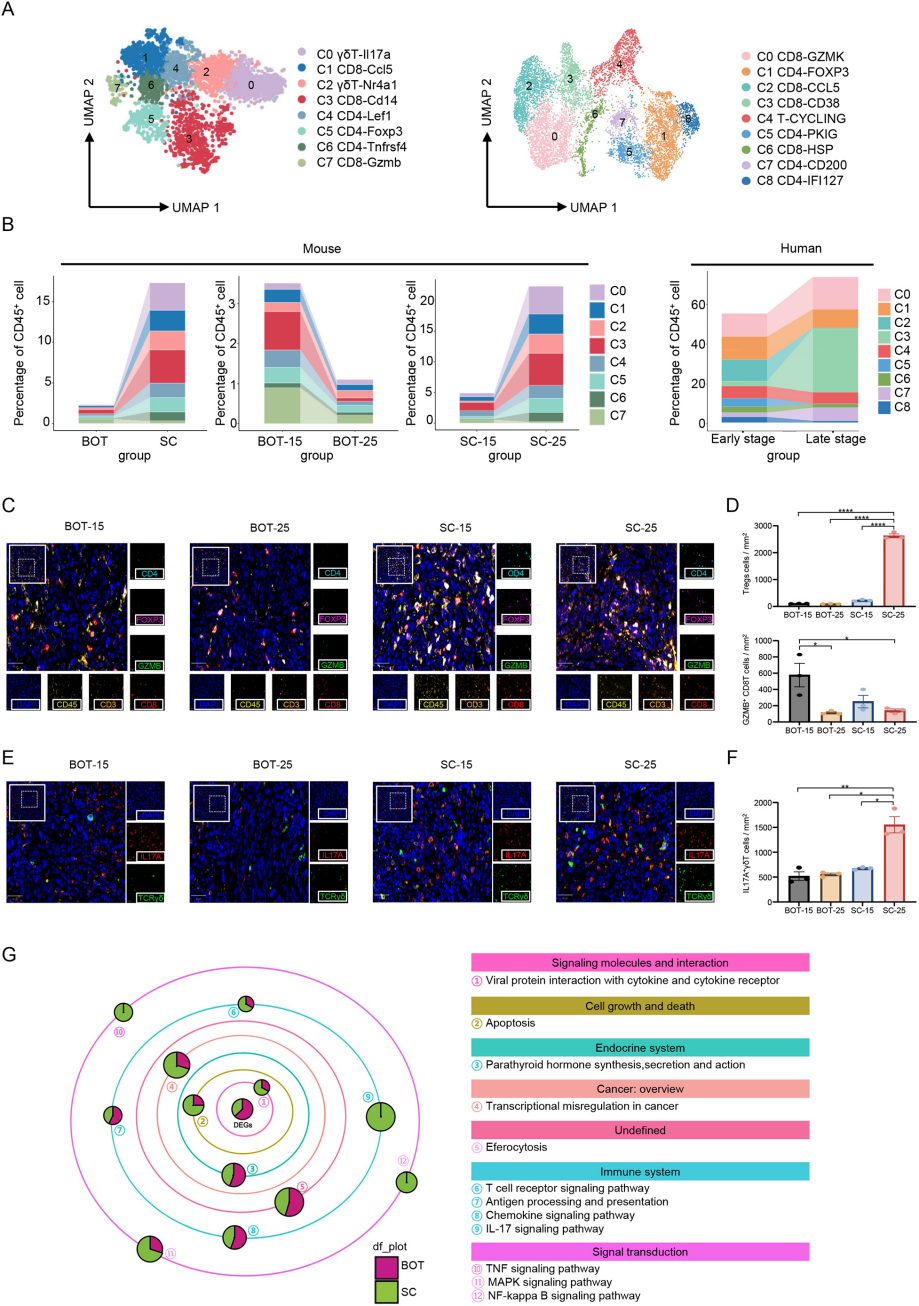
**Distinct T-cell dynamics between murine models and human HPV-positive OPSCC during tumor progression.** (A) Uniform manifold approximation and projection (UMAP) plots of T cells identified in the SC murine model (left) and from human HPV-positive OPSCC (right), showing the formation of eight and nine subclusters, respectively. Each dot represents an individual cell, color-coded by cluster number. (B) Left: histograms showing the percentage changes of T-cell subclusters in CD45-positive (CD45^+^) cells in different murine models (BOT vs SC) as well as in BOT and SC models at different stages (i.e. 15 vs 25 days post implantation). Right: histogram showing the percentage changes of T-cell subclusters among CD45^+^ cells at different clinical stages in human HPV-positive OPSCC. (C) Representative merged immunofluorescence images of BOT and SC tumors harvested at days 15 and 25 post implantation (BOT-15 and BOT-25, and SC-15 and SC-25, respectively), stained for effector T (Teff) cells – which stain positive for CD45 (yellow), CD3 (orange), CD8 (red) and GZMB (green) – as well as for regulatory T (Treg) cells – which stain positive for CD45 (yellow), CD3 (orange), CD4 (cyan) and FOXP3 (pink). Nuclei were stained with DAPI (blue). A panoramic view is shown in the top-left panel (scale bars: 100 μm), with a region of interest (ROI) boxed. This ROI is shown magnified (×5) in the bottom-right corner, which depicts the corresponding merged image. Scale bars for the magnification: 50 μm. (D) Treg cells (top) and Teff cells (bottom) per mm² tumor area for each group. Data are presented as the mean±s.e.m. (five fields/mouse; *n*=3 mice/group). (E) Representative merged immunofluorescence images of BOT-15, BOT-25, SC-15 and SC-25 tumors harvested at days 15 and day 25 post implantation, stained for IL17A-positive γδ T cells (IL17A^+^TCRγδ^+^). A panoramic view is shown in the top-left panel (scale bars: 100 μm), with a region of interest (ROI) boxed. This ROI is shown magnified (×5) in the bottom-right corner, which depicts the corresponding merged image. Scale bars for the magnification: 50 μm. (F) IL17A^+^ γδ T cells per mm² tumor area for each group. Data are presented as the mean±s.e.m. (five fields/mouse× three mice/group). (G) Galaxy diagram showing differentially regulated genes in T cells from BOT and SC models, categorized by known or predicted function(s), literature and sequence similarity. Circle sizes are proportional to the number of differentially expressed genes (Significance <0.05). Statistical significance of data shown in panels D and F was determined by two-way ANOVA with Tukey's post-hoc test: *P<0.05, **P<0.01 and ****P<0.0001.

Given that intra-tumoral T cells are critical players in anti-tumor immunity (Prokhnevska et al., 2023; Yao et al., 2024), we isolated T cells from the TIME of murine models and human HPV-positive OPSCC cells for further analysis. A total of eight and nine T-cell subclusters were identified in murine models and human HPV-positive OPSCC, respectively (Fig. 2A and Tables S3-S4). In general, T-cell infiltration was much higher in the SC models than in the BOT models (∼15%). Additionally, the proportions of effector memory T cells and effector CD8-positive (CD8^+^) T cells declined substantially during tumor progression in BOT models, whereas they increased in SC models. These findings suggested that the establishment of anti-tumor immunity is more efficient in SC than in BOT models. Nevertheless, we also observed rapid accumulation of IL17A-positive (IL17A^+^) γδ T cells and Treg cells in the TIME of SC models (Fig. 2B). Both were abundant in the skin and had previously been reported to facilitate tumor immune escape and instigate resistance to immunotherapy (Du et al., 2022; Edwards et al., 2023; Lainé et al., 2021; You et al., 2024). Meanwhile, our mIHC experiments also verified that infiltration of effector CD8^+^ T cells declined remarkably during tumor progression in BOT models, whereas statistically significant accumulation of IL17A^+^ γδ T cells and Treg cells was detected in the TIME of SC models (Fig. 2C-F).

Consistently, although functional analysis demonstrated the enrichment of anti-tumor pathways, including T-cell activation, and TNF- and NF-κB signaling pathways in T cells from SC models, they also exhibited significant enrichment in pro-tumor IL-17 signaling pathways (Fig. 2G). Taken together, intra-tumoral T-cell analysis revealed higher infiltration and increased proportions of effector T cells in SC models than in BOT models, along with an accumulation of IL17A^+^ γδ T cells and Treg cells in SC models, suggesting a more robust but complex anti-tumor immune environment involving both anti-tumor and pro-tumor pathways. However, the majority of T cells in the TIME of human HPV-positive OPSCC showed cytotoxic potential – including effector CD8^+^ T cells and exhausted CD8^+^ T cells – that further increased during tumor progression (Fig. 2B and Fig. S2). The observed increase in T-cell proportions in late-stage human tumors (∼75% vs ∼50% in early stage) aligns directly with the progressive immune infiltration in the SC mouse model. While this trend might reflect cumulative antigen exposure over time, which is consistent with established immunobiology of chronic tumor evolution (Rossa and D'Silva, 2019; Chaves et al., 2023), our cross-sectional human data cannot establish temporal causality. Future longitudinal studies are needed to validate these dynamics.

Collectively, these findings indicate that, although SC murine models exhibit increased infiltration of effector T cells together with accumulation of IL17A^+^ γδ T cells and Treg cells compared to BOT models, human HPV-positive OPSCCs show a predominant and stage-dependent increase in cytotoxic T cells, which might reflect cumulative antigen exposure over time.

### Distinct myeloid cell dynamics in murine models and human HPV-positive OPSCC during tumor progression

**Fig. 3.**
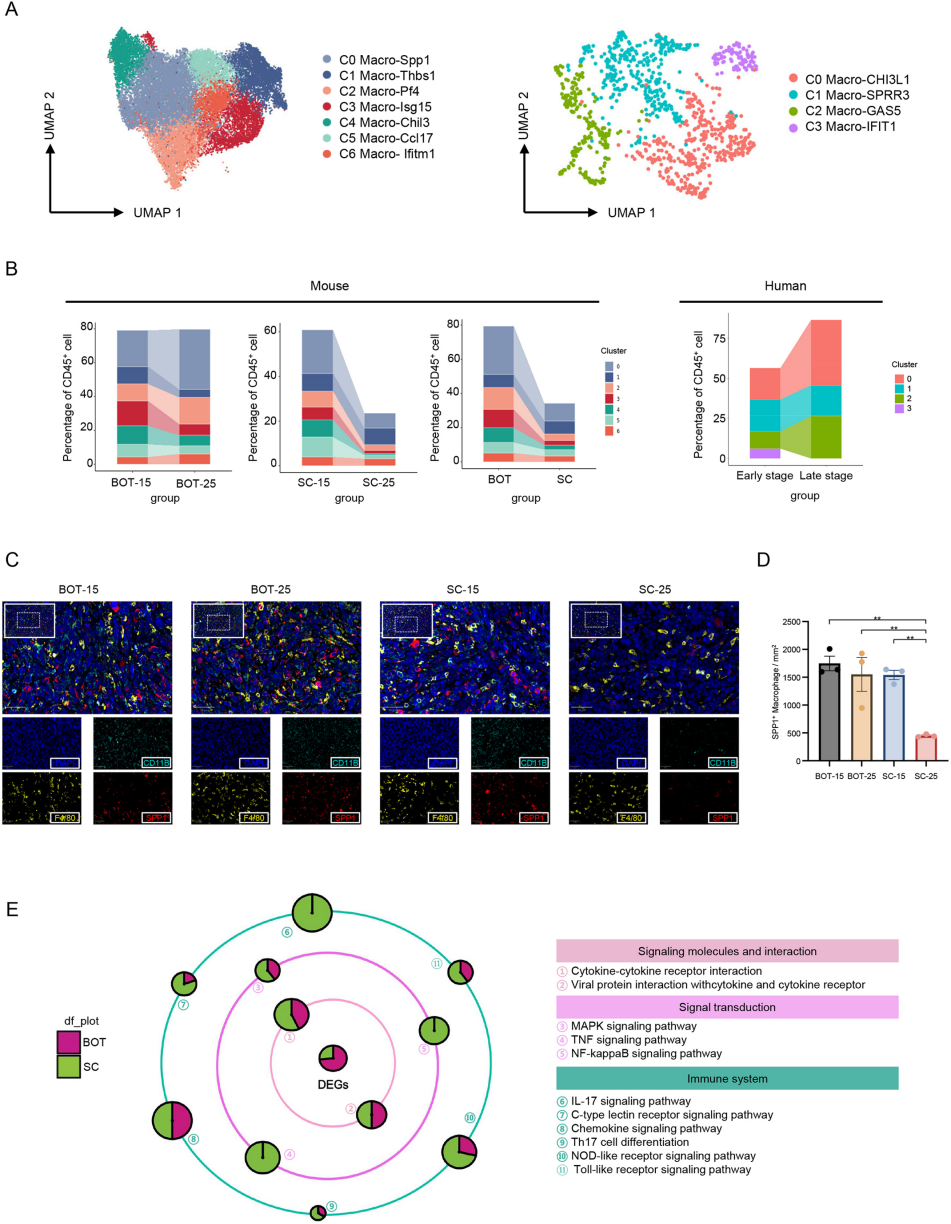
**Distinct macrophage dynamics between murine models and human HPV-positive OPSCC during tumor progression.** (A) Uniform manifold approximation and projection (UMAP) plot (left) of macrophages identified in the murine model (left) and from human HPV-positive OPSCC (right), showing showing the formation of seven and four subclusters, respectively. Each dot represents an individual cell, color-coded by cluster number. (B) Histogram (left three) depicting the percentage changes of macrophage subclusters among CD45-positive (CD45^+^) cells between different murine models (BOT vs SC) and different stages (early vs late) in SC and BOT models. Histogram (right) depicting the percentage changes of macrophage subclusters among CD45^+^ cells at different clinical stages in human HPV-positive OPSCC. (C) Representative merged immunofluorescence images of SC and BOT tumors harvested at days 15 and 25 post implantation (BOT-15 and BOT-25, and SC-15 and SC-25, respectively), stained for SPP1-positive (Spp1^+^) macrophages – i.e. staining against CD11B (cyan), F4/80 (yellow) and SPP1 (red). Nuclei were stained with DAPI (blue). A panoramic view is shown in the top-left panel (scale bars: 100 μm), with a region of interest (ROI) boxed. This ROI is shown magnified (×5) in the bottom-right corner, which depicts the corresponding merged image. Scale bars for the magnification: 50 μm. (D) Bar graph showing the number of SPP1^+^ macrophages per mm² tumor area for each group. Error bars present the mean±s.e.m. (five fields/mouse; *n*=3/group). Statistical significance was determined by two-way ANOVA with Tukey's post-hoc test: **P<0.01. (E) Galaxy diagram showing the differentially regulated genes in macrophages obtained from BOT and SC models, categorized by known or predicted function(s), literature and sequence similarity. Circle sizes are proportional to the number of differentially expressed genes (*P*<0.05).

The heterogeneity of myeloid cells with immunosuppressive functions has been well-characterized in various types of human and murine tumor (Cheng et al., 2021; Mantovani et al., 2021). Although SC and BOT models both showed a more myeloid-biased TIME, the proportion of myeloid populations, especially of macrophages, was remarkably higher in the TIME of BOT models than of SC models. Moreover, macrophages obtained from murine models seemed to have increased polyfunctionality than their counterparts obtained from human tumors (Fig. 3A,B, Fig. S3A-C, Tables S5 and S6). Specifically, we found macrophages positive for Spp1 and Pf4 (Spp1^+^ and Pf4^+^, respectively) being the predominant types of immune cell in both models, i.e. polarized towards an M2 phenotype, and accompanied by a potent capacity for phagocytosis and complement activation (Fig. 3B-E and Fig. S3B-E). Nevertheless, as the tumor progressed, these two M2 macrophage populations rapidly accumulated in the TIME of BOT models but diminished in the TIME of SC models. Meanwhile, our mIHC experiments also verified a statistically significant increase of M2 macrophage infiltration in BOT models, whereas the opposite trend was observed in TIME of SC models (Fig. 3C,D). Given that both Spp1^+^ macrophages and Pf4^+^ macrophages have previously been reported to inhibit the cytotoxic potential of CD8^+^ T cells and facilitate tumor evasion (Sathe et al., 2023; Zhang et al., 2024; Liu et al., 2024), our findings suggested that, during tumor progression, macrophages from BOT models have a gradually enhanced suppressive TIME, and that this suppressive potential gradually diminishes in the TIME of SC models.

Consistently, functional analysis demonstrated less enrichment of pathways related to positive regulation of innate immunity, including Toll-like receptor signaling pathways and TNF signaling pathways in macrophages from BOT models (Fig. 3E). By contrast, macrophages from human HPV-positive OPSCC are less abundant and heterogeneous, and their proportions remain relatively stable during tumor progression.

Furthermore, neutrophil infiltration appeared to be more abundant in the TIME of SC compared with BOT models (Fig. S4A-C, Table S7). Specifically, we found that neutrophils positive for Ccl3, Myo1e and Mmp8 (Ccl3^+^, Myo1e^+^ and Mmp8^+^, respectively) being the predominant types of immune cell in both models. Ccl3^+^ and Mmp8^+^ neutrophils were characterized by IFN-stimulated gene signatures and significant functional enrichment in pathways related to TNF signaling and neutrophil extracellular trap (NET) formation, whereas Myo1e^+^ neutrophils exhibited functional enrichment in angiogenesis and RAS protein signal transduction (Fig. S4F-H). As the tumor progressed, pro-inflammatory neutrophils (i.e. Ccl3- and Mmp8^+^ neutrophils) infiltrated the TIME of the SC models, whereas angiogenic neutrophils (i.e. Myo1e^+^ neutrophils) remained the predominant type of immune cell in the TIME of the BOT models. And these findings were also verified by mIHC experiments (Fig. S4D-E). Collectively, SC models exhibit higher neutrophil infiltration with a predominance of pro-inflammatory phenotypes, while BOT models are dominated by angiogenic neutrophils, indicating the distinct role that neutrophils play in the TIME of SC and BOT models.

Similarly, DC infiltration was more abundant in the TIME of SC than in that of BOT models (Fig. S5A-C and Table S8). Notably, we identified a DC subcluster that is predominantly positive for Fscn1 (Fscn1^+^), with potent migratory potential and functional enrichment in pathways related to T-cell proliferation, which was defined as migratory DCs (Fig. S5D-F). Consistent with the results of mIHC experiments, statistically significant declines in DC numbers were observed both in the TIME of SC models and BOT models during tumor progression (Fig. 1G, L), suggesting DC function is impaired during progression of HPV-positive OPSCC. However, a remarkable plasticity of DCs has been previously reported (Kvedaraite and Ginhoux, 2022; Li et al., 2023), so it remained to be further investigated that whether these DCs were positively or negatively associated with T-cell proliferation.

Collectively, our findings support the evidence that myeloid cells from BOT models have a gradually enhanced suppressive TIME during tumor progression, which is distinct from the TIME of SC models and human samples.

### BOT and SC models exhibit distinct responses to canonical ICBs

**Fig. 4.**
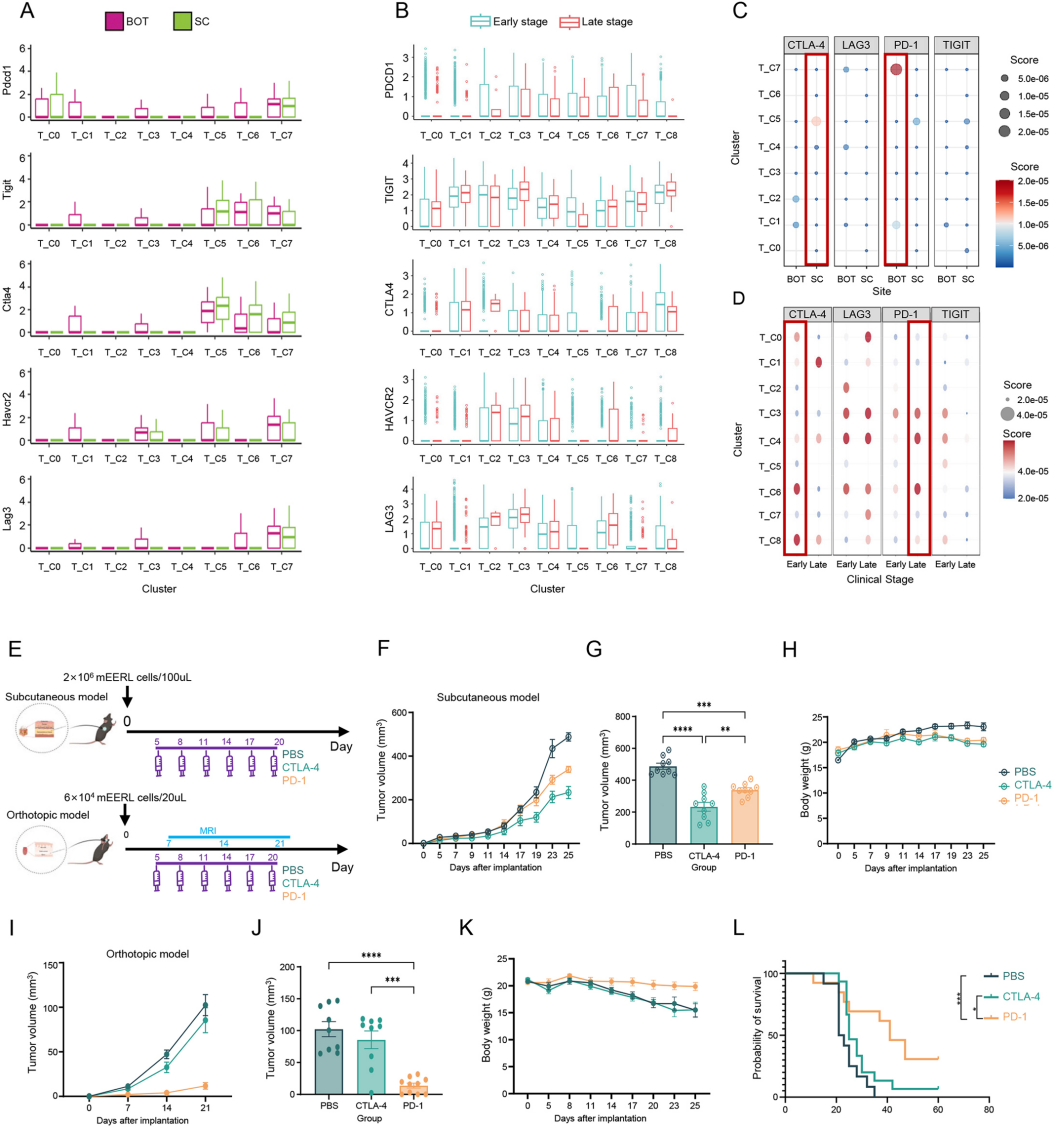
**BOT and SC models exhibited differential responses to canonical immune checkpoint blockades (ICBs).** (A) Box plots showing the expression patterns of levels of canonical inhibitory receptor genes *Pdcd1*, *Tigit*, *Ctla4*, *Havcr2* and *Lag3* in T-cell subclusters from murine models. (B) Box plots showing the expression patterns of levels of canonical inhibitory receptor genes *PDCD1*, *TIGIT*, *CTLA4*, *HAVCR2* and *LAG3* in T-cell subclusters from human HPV-positive OPSCC. (C) Dot plot showing the perturbation scores (perb_score) for various T-cell subclusters in BOT and SC models in response to different ICBs (CTLA4, LAG3, PDCD1 and TIGIT). (D) Dot plot showing the perturbation scores (perb_score) for various T-cell subclusters in human HPV-positive OPSCC in response to different ICBs (CTLA4, LAG3, PDCD1 and TIGIT). (E) Schematic of therapeutic study design. The therapeutic study design employed mEERL cell tumor models via subcutaneous (flank) or orthotopic (base of tongue) implantation, with both cohorts receiving intraperitoneal ICB treatments every 3 days starting on day 5 post implantation: anti-PDCD1 at 200 μg/dose and anti-CTLA4 at 100 μg/dose. (F,G) Tumor growth of SC models following treatment (*n*=9). (H) Body weight change of SC models following treatment (*n*=9). (I,J) Tumor growth of BOT models following treatment (*n*=9). (K) Body weight change of BOT models following treatment (*n*=9). (L) Kaplan–Meier survival curves for BOT models following treatment (*n*=9). ICBs, immune checkpoint blockades. Statistical significance of data shown in panels F, G, I and J was determined by two-way ANOVA with Tukey's post-hoc test; for data shown in panel L, Log-rank test was used. *P<0.05, **P<0.01, ***P<0.001 and ****P<0.0001. Data are presented as the mean+s.e.m. (A,B) or mean±s.e.m. (F-K).

Given that ICB is the most used immunotherapy for reversing the immunosuppressive TIME of OPSCC (Roof and Yilmaz, 2023), we further investigated the extent to which our models can recapitulate human responses to canonical ICBs. We first explored the expression levels of canonical inhibitory receptor genes in T cells (the major responders to ICBs) in both murine models and human samples. The results demonstrated similar expression patterns and comparable expression levels of these inhibitory receptor genes between murine models and human samples (Fig. 4A,B). As expected, the majority of inhibitory receptor genes were expressed in effector CD8^+^ T cells, exhausted CD8^+^ T cells and Treg cells. Accordingly, we then matched drug-responsive cell types corresponding to the currently commercialized types of ICB (Fig. 4C,D). Specifically, exhausted CD8^+^ T cells, effector CD8^+^ T cells and Treg cells from early-stage human samples exhibited remarkable responses to CTLA4 blockade, showing similarity with SC models, which were characterized by a Treg-centered response to CTLA4 blockade. BOT models and late-stage human samples responded similarly to blockage of programmed cell death protein 1 (PDCD1, also known as PD-1), with cytotoxic CD8^+^ T-cell subclusters being the main drug-responsive cell type. Nevertheless, both the SC and BOT models were predicted to respond poorly to the LAG3 and TIGIT blockade, which was different from human responses. Moreover, as previously demonstrated by us, SC models show a rapid accumulation of Treg cells during tumor progression; Moreover, as described earlier, SC models might be more reasonable to apply CTLA4 blockade in the late stage. However, BOT models were characterized by a gradual elimination of effector CD8^+^ T cells, suggesting the need for early PDCD1 blockade.

To functionally test our spatial transcriptomics-based predictions of anatomical context-dependent ICB responses, we conducted *in vivo* therapeutic trials in both orthotopic (*n*=9) and subcutaneous (*n*=9) tumor-bearing mice (Fig. 4E-L, Fig. S6A,B). As expected, anti-CTLA4 monotherapy induced significant tumor regression in subcutaneous models, whereas orthotopic tumors exhibited primary resistance to CTLA4 blockade. By contrast, anti-PDCD1 treatment achieved disease control in orthotopic tumors, but showed minimal efficacy in subcutaneous models. This diametric response pattern aligns with our computational drug sensitivity profiling and underscores the critical influence of tumor microenvironmental atlases on therapeutic vulnerability.

Collectively, BOT and SC models exhibited distinct responses to canonical ICBs, with BOT models mirroring the early-stage human response to PDCD1 blockade and SC models mirroring the late-stage human response to CTLA4 blockade.

## DISCUSSION

In this study, we comprehensively characterized immune cell heterogeneity and functional dynamics during tumor progression in BOT and SC murine models of HPV-positive OPSCC. Our findings revealed distinct immunosuppressive mechanisms and drug responsiveness between these models, highlighting both their strengths and limitations in recapitulating the key features of human HPV-positive OPSCC.

Consistent with previous studies, we observed that both BOT and SC models are dominated by myeloid immune cells, particularly macrophages and neutrophils, both of which are known to drive tumor progression and resistance to immunotherapies through immunosuppressive signaling pathways (Zhang et al., 2024; Mantovani et al., 2021). However, we detected significant differences in T-cell dynamics between the two models, with SC tumors displaying a progressive increase in T-cell infiltration – particularly of effector CD8^+^ T cells that are central to anti-tumor immunity (Yao et al., 2024). By contrast, BOT tumors exhibited a steady decline in T-cell population – particularly in that of cytotoxic T cells – suggesting that the BOT model more closely mimics the immune desertification seen in advanced, immune-excluded human tumors (Quah et al., 2023). While our scRNA-seq analysis revealed dynamic immune shifts in mouse models (Fig. 1H), we acknowledge a substantial quantitative discrepancy: human OPSCC samples exhibited dominant T-cell infiltration (50–75%), whereas baseline T-cell presence was insufficient in both SC (<5%) and BOT (<20%) mouse models. This divergence likely stems from several key factors. First, species-specific immunobiology, i.e. human tumors evolve in lymphoid-rich microenvironments (e.g. tonsillar crypts) with chronic antigen exposure, while both orthotopic and subcutaneous mouse models lack this anatomical context (Chaves et al., 2023; Kono et al., 2022). Second, methodological differences in tissue acquisition, i.e. human samples preserved stromal architecture through rapid cryopreservation, whereas mouse dissociation protocols may damage or lead to the loss of fragile stromal populations (Guerin et al., 2020; Zhou et al., 2024). In addition, the observed ‘increase’ in the proportion of T-cells in late-stage human samples is consistent with previous findings; however, whether this primarily reflects a reduction in B-cell frequency or an expansion in the total number of T-cells remains to be further investigated. Importantly, although neither model fully replicates the high-infiltrate human phenotype, their value lies in capturing progressive trends. In the SC model, the increasing T-cell influx mirrors immunoediting dynamics in a subset of human cases, while the BOT model exemplifies T-cell exclusion mechanisms that are relevant in ‘immune-desert’ or ‘cold’ tumors, i.e. tumors with insufficient numbers or lack of T cells (Rossa and D'Silva, 2019). Future studies should prioritize orthotopic engraftment and/or humanized systems to better recapitulate the lymphoid−tumor crosstalk in humans.

The distinct myeloid landscape between BOT and SC models further underscores the complexity of the TIME in HPV-positive OPSCC. BOT tumors are characterized by a substantial accumulation of M2-polarized macrophages, which are associated with poor clinical outcomes owing to their ability to inhibit T-cell activation and promote tumor growth (Nasir et al., 2023; Revel et al., 2022; Toledo et al., 2024). Additionally, BOT models exhibit persistent infiltration of angiogenic neutrophils, further reinforcing a suppressive TIME (Antuamwine et al., 2023; Giese et al., 2019; Horvath et al., 2024). By contrast, SC tumors showed a gradual shift toward pro-inflammatory neutrophils, which could partially explain the higher T-cell infiltration observed in these tumors. These findings suggest that the BOT model more accurately reflects the late-stage immunosuppressive environment observed in human OPSCC, in which accumulation of pro-tumoral M2 macrophages and angiogenic neutrophils contributes to immune evasion and resistance to ICBs.

A key finding of our study is the distinct response of BOT and SC models to PDCD1 blockade. Unexpectedly, although SC models exhibited a more dynamic and responsive T-cell landscape than BOT models, BOT models responded to PDCD1 blockade in a way similar to that in human samples, with effector CD8^+^ T-cell subclusters being the main drug-responsive type of immune cell. This inconsistency might be due to the fact that, despite the higher T-cell infiltration in SC models, we observed a concurrent rise in pro-tumor T-cell subsets, including Tregs and IL17A^+^ γδ T cells, both of which are associated with immune suppression and resistance to ICBs (Lainé et al., 2021; You et al., 2024; Ma et al., 2014; Du et al., 2022). This suggests that, although the SC model might initially respond to PDCD1 blockade, the presence of suppressive T-cell populations limits the durability of the response, making the SC model an important system for studying the mechanisms of resistance to PDCD1 therapy.

However, our study also has several limitations. First, although we employed the use of mEERL – the most widely validated HPV-positive OPSCC cell line – it fails to completely capture the molecular heterogeneity and immune microenvironment diversity of clinical HPV-positive OPSCCs, potentially limiting conclusions about immune-regulatory mechanism to cell line-specific biology rather than universal principles. Second, although scRNA-seq enabled deep characterization of immune subsets, inherently low-abundance cell populations – particularly macrophages, DCs and neutrophils in human OPSCC specimens – were captured in limited numbers. This scarcity unavoidably constrains cluster resolution and diversity metrics, potentially exaggerating apparent reductions in transcriptional heterogeneity. Future studies would benefit from targeted enrichment strategies or spatial transcriptomics to resolve low-frequency states of rare cell types.

### Conclusion

**Fig. 5.**
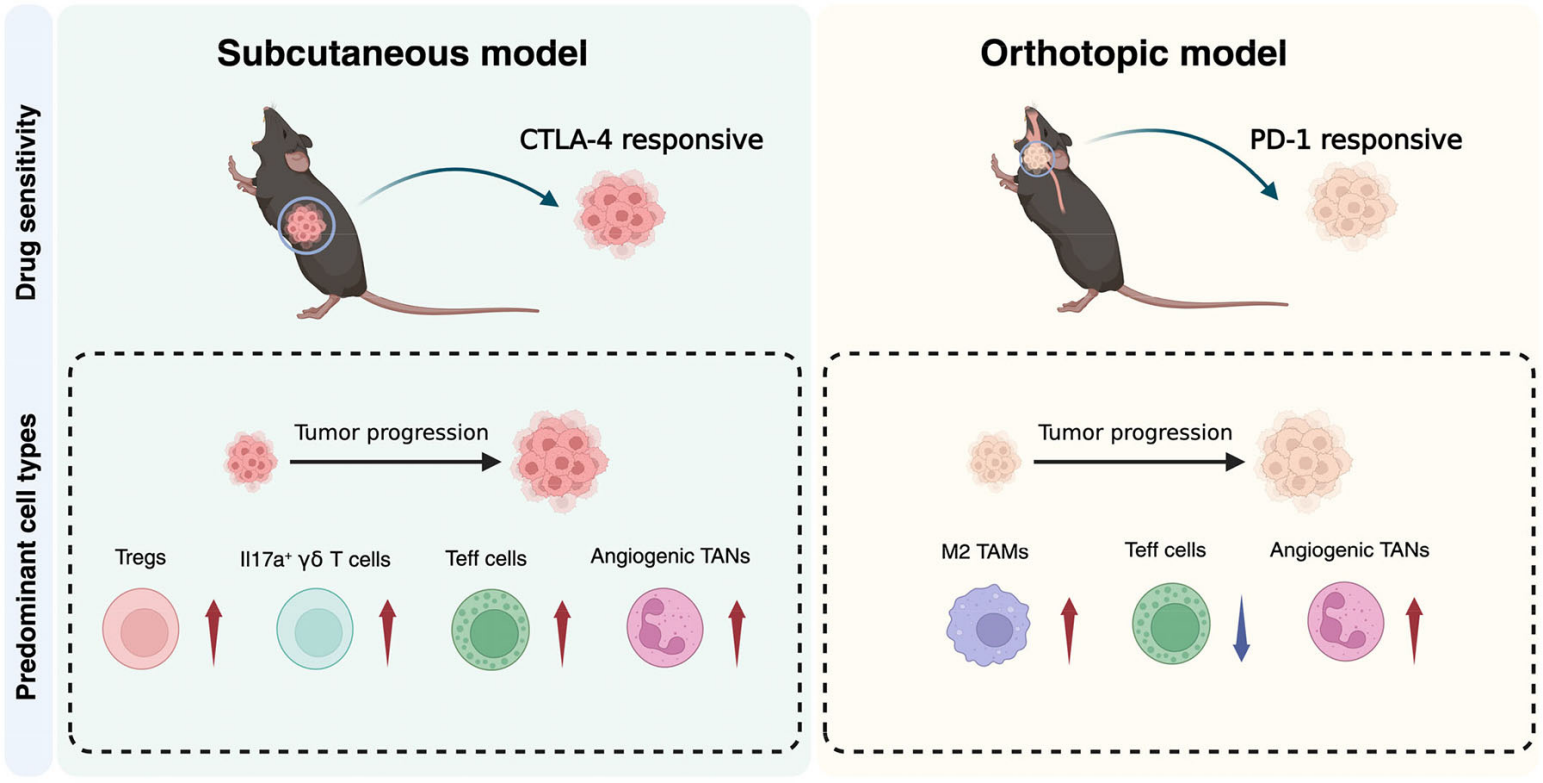
**Schematic representation of immune microenvironment dynamics and drug responsiveness in BOT and SC murine models of HPV-positive OPSCC.** Schematic of the distinct immune microenvironments and drug sensitivities observed in subcutaneous (SC) and orthoptic (BOT) murine models of HPV-positive OPSCC across tumor progression stages. In the SC model, tumor progression is accompanied by increased infiltration (upward arrows) of Tregs, IL17A^+^ γδ T cells, Teff cells and pro-angiogenic tumor-associated neutrophils (TANs). This model is selectively responsive to CTLA4 blockade. By contrast, the BOT model exhibits progressive immune suppression dominated by M2 tumor-associated macrophages (TAMs) and angiogenic TANs, together with reduced infiltration (downward arrows) of Teff cells. The BOT model is responsive to PDCD1 blockade. Created in BioRender by Mao, M., 2025. https://BioRender.com/cduzhqq. Republished with permission.

In conclusion, our study demonstrates that, although both BOT and SC murine models provide valuable insights into the immune landscape of HPV-positive OPSCC, they exhibit distinct immunological dynamics that should be carefully considered when selecting models for preclinical testing (Fig. 5). The BOT model, with its strong resemblance to the suppressive myeloid environment and immune desertification seen in advanced human tumors, might be more suitable when evaluating therapies to target late-stage diseases. The SC model, by contrast, with higher T-cell infiltration and dynamic immune landscape, might be more appropriate for investigating early stage immunotherapies. Together, these findings advance our understanding of the HPV-positive OPSCC TIME and provide a foundation for optimizing preclinical models to improve therapeutic outcomes.

## MATERIALS AND METHODS

### Animals

Male C57Bl/6J mice (6–8 weeks old) were purchased from SPF (Beijing) Biotechnology Co., Ltd.). All the mice were housed under specific pathogen-free (SPF) conditions with *ad libitum* access to food and water. Animal experiments were conducted in strict accordance with the guidelines of the Animal Ethics Committee of West China Hospital (Approval No.: 20220511001) and the ethical standards outlined in the Guide for the Care and Use of Laboratory Animals by the China Association for Laboratory Animal Care. The euthanasia procedures followed institutional protocols. All procedures were designed and reported in compliance with the ARRIVE guidelines (Animal Research: Reporting of In Vivo Experiments).

### Cell lines

The mEERL cell line, originally derived from the oropharyngeal epithelium of C57BL/6J mice (Zandberg et al., 2021; Newton et al., 2019), is commonly used in HPV-positive syngeneic mouse models. The mEERL cells were maintained in PriGrow IV medium (cat. no.: TM004, Applied Biological Materials) at 37°C in a humidified incubator under 5% CO₂, as previously described (Qiu et al., 2023). Routine mycoplasma tests were conducted throughout the study and all results were negative.

### *In vivo* tumor challenge

For the orthotopic model, 6×10⁴ cells suspended in 20 μl sterile phosphate-buffered saline (PBS) were injected into the base of the tongue. The mice were observed regularly and euthanized with a weight loss of 20% or more from their initial body weight. For the subcutaneous tumor model, 2×10⁶ cells were suspended in 100 μl sterile PBS and injected subcutaneously into the right flank of each mouse. Tumor dimensions were recorded every 2–3 days using Vernier calipers and tumor volume were calculated using the formula: volume=π/6×length×width². Mice were sacrificed when tumor volumes surpassed 1000 mm³ or ulceration developed. All orthotopic mice reached humane endpoints necessitating euthanasia by day 40, whereas subcutaneous cohorts were electively sacrificed at study termination (day 40) without reaching clinical endpoints.

### Magnetic resonance imaging (MRI)

Mice underwent imaging on days 7, 14, 21 and 25 after the tumor challenge using a Bruker 7.0T animal MRI scanner. Tumor volumes were calculated in three dimensions using the 3D Slicer software.

### Tissue collection and single-cell suspension preparation

To characterize tumor-infiltrating lymphocytes (TILs), mice were sacrificed at the time points indicated in the results section. Tumors from the tongue and flank were harvested and processed according to established protocols (Ferlay et al., 2019). The tissues were then incubated on a rocker at 160 rpm for 15-40 min at 37°C. After digestion, the samples were filtered through a 70-μm cell strainer to eliminate any remaining cell clumps and centrifuged at 500 ***g*** for 5 min. Immune cells were stained at a concentration of 1×10⁶ cells/ml with anti-CD45 antibody (clone 30-F11, BioLegend, cat. no.: 103130) for 30 min at 4°C. After washing, the cells were suspended in 0.04% bovine serum albumin (BSA). For dead cell discrimination, 7-AAD viability staining solution (BioLegend, cat. no.: 420404) was added 5 min before flow cytometry. CD45^+^ cells were sorted using a FACS Aria II Cell Sorter (BD Biosciences), with post-sort purity routinely exceeding 95%. Unless otherwise specified, all subsequent steps were performed on ice or at 4°C.

### Multiplex immunohistochemistry (mIHC)

To quantitatively characterize the immune landscape of TILs across different stages and anatomical sites, multiplex immunohistochemistry (mIHC) was performed on formalin-fixed paraffin-embedded (FFPE) tumor tissues (*n*=3 mice per group). A total of 12 tumor samples were sectioned at 4 μm thickness and subjected to mIHC using the Opal Polaris 7-color system (Akoya Biosciences).

Following standard deparaffinization and hydration, tissue sections underwent heat-induced epitope retrieval in citrate buffer (pH 6.0) using a microwave-based protocol. Endogenous peroxidase activity was quenched and sections were blocked in a buffer containing 1% BSA, 5% FBS and 0.2% Triton X-100. Sequential rounds of antibody staining were performed using primary antibodies targeting immune cell markers including CD45 (CST, #70257, 1:200), CD3 (CST, #78588, 1:200), CD8 (Abcam, #ab217344, 1:100), GZMB (CST, #44153, 1:100), CD4 (CST, #25229, 1:200), FOXP3 (CST, #12653, 1:200), TCRγδ (STEMCELL, #60104AD.1, 1:200), IL-17a (CST, #70257, 1:100), CD19 (Abcam, #ab245235, 1:1000), CD11b (Abcam, #ab133357, 1:1000), F4/80 (CST, #30325, 1:200), SPP1 (CST, #88742, 1:100), CD11c (CST, #97585, 1:100), Ly-6G (CST, #87048, 1:100) and CCL3 (CST, #85270, 1:200), according to panel design for each slide. Antibodies were diluted in blocking buffer and incubated overnight at 4°C.

Primary antibodies were visualized using HRP-conjugated secondary detection reagents and tyramide signal amplification with fluorophores from the Opal 7-color kit, with the following fluorophore-channel assignments and antibody dilutions: CD45 (Opal 520/570/620, 1:200), CD3 (Opal 480, 1:200), CD8 (Opal 690, 1:100), GZMB (Opal 520, 1:100), CD4 (Opal 570, 1:200), FOXP3 (Opal 780, 1:200), TCRγδ (Opal 520, 1:200), IL-17a (Opal 690, 1:100), CD19 (Opal 780, 1:1000), CD11b (Opal 480, 1:1000), F4/80 (Opal 570, 1:200), SPP1 (Opal 690, 1:100), CD11c (Opal 620, 1:100), Ly-6G (Opal 620, 1:100) and CCL3 (Opal 780, 1:200). Nuclei were counterstained with spectral DAPI.

Stained slides were scanned using the Vectra^®^ Polaris™ multispectral imaging system. Multichannel images were unmixed and analyzed using QuPath software (version 0.5.1), enabling cell-type classification, spatial mapping and quantitative assessment of immune cell infiltration and location across tumor regions.

### Single-cell RNA sequencing library preparation and sequencing

Single-cell RNA sequencing (scRNA-seq) libraries were generated following the manufacturer's protocol (10× Genomics, Next GEM Single Cell 5′ GEM Kit v2, 1000244). Sequencing was performed using a NovaSeq 6000 system, achieving an average depth of 30,000 reads per cell.

### scRNA-seq library pre-processing, quality-control and cell type annotation

Raw scRNA-seq data were processed using Cell Ranger v7.0.1 (10× Genomics, http://10xgenomics.com/), with reads aligned to the mm10 reference genome (refdata-gex-mm10-2020-A). Sequencing quality was assessed using metrics, such as valid cell barcode rates, transcriptome mapping efficiency and the proportion of Q30 bases. The samples were then merged into a unified gene/barcode matrix for further analyses. Data pre-processing was conducted using Seurat (https://satijalab.org/seurat/) (v4.3.1), with clusters containing low total UMI counts or a high proportion of mitochondrial transcripts removed. Doublet cells were excluded using the scDblFinder package (v1.16.0; https://bioconductor.org/packages/release/bioc/html/scDblFinder.html).

To correct for batch effects, the harmony algorithm (v0.1.0) was applied. Clustering was performed with Seurat's FindCluster function and dimensionality reduction was achieved using the RunTSNE function (reduction=‘harmony,’ dims=1:30). Differentially expressed genes (DEGs) for each cluster were identified using FindAllMarkers (https://satijalab.org/seurat/reference/findallmarkers) (min. pct=0.25, logfc.threshold=0.25). Cell type annotation was based on DEGs and the expression of known marker genes.

### Human scRNA-seq data collection

scRNA-seq data from four human OPSCC cases were obtained from publicly available datasets [GSE182227 (https://www.ncbi.nlm.nih.gov/geo/query/acc.cgi?acc=GSE182227) (Puram et al., 2023), GSE181919 (https://www.ncbi.nlm.nih.gov/geo/query/acc.cgi?acc=GSE181919) (Choi et al., 2023) and GSE226620 (https://www.ncbi.nlm.nih.gov/geo/query/acc.cgi?acc=GSE226066) (Cha et al., 2024)], as detailed in Table S9. The use of these datasets was reviewed and approved by the Biomedical Research Ethics Committee of West China Hospital (approval number: 2022-1174). The data are available under open access and were accessed in compliance with the terms of use specified by the original data providers. Quality control parameters and cell type annotation procedures were identical to those described earlier for the mouse scRNA-seq data were applied to these human datasets to ensure consistency in data processing and analysis.

### Functional annotation analyses

Kyoto Encyclopedia of Genes and Genomes (KEGG) and Gene Ontology (GO) enrichment analyses of DEGs between groups as well as target genes from cell-to-cell communication were performed using the clusterProfiler package (v4.10.0, https://github.com/GuangchuangYu/enrichment4GTEx_clusterProfiler). Gene expression clustering and visualization were performed using the ClusterGVis package (v0.1.1, https://github.com/junjunlab/ClusterGVis) with default parameters.

### Scoring of gene expression signatures

Signature scores for individual cells were computed using Seurat's AddModuleScore function. Macrophage signatures were calculated for antigen presentation (*HLA-DMA*, *HLA-DMB*, *HLA-DOA*, *HLA-DOB*, *HLA-DPA1*, *HLA-DPB1*, *HLA-DQA1*, *HLA-DQA2*, *HLA-DQB1*, *HLA-DQB2*, *HLA-DRA*, *HLA-DRB1*, *HLA-DRB5*), complement activation (*C1QC*, *C1QB*, *C1QA*, *APOE*, *APOC1*), angiogenesis (*CCND2*, *CCNE1*, *CD44*, *CXCR4*, *E2F3*, *EDN1*, *EZH2*, *FGF18*, *FGFR1*, *FYN*, *HEY1*, *ITGAV*, *JAG1*, *JAG2*, *MMP9*, *NOTCH1*, *PDGFA*, *PTK2*, *SPP1*, *STC1*, *TNFAIP6*, *TYMP*, *VAV2*, *VCAN*, *VEGFA*) and phagocytosis (*MRC1*, *CD163*, *MERTK*, *C1QB*). M1 and M2 macrophage signatures were derived from M1-associated (*CCL5*, *CCR7*, *CD40*, *CD86*, *CXCL9*, *CXCL10*, *CXCL11*, *IDO1*, *IL1A*, *IL1B*, *IL6*, *IRF1*, *IRF5*, *KYNU*) and M2-associated genes (*CCL4*, *CCL13*, *CCL18*, *CCL20*, *CCL22*, *CD276*, *CLEC7A*, *CTSA*, *CTSB*, *CTSC*, *CTSD*, *FN1*, *IL4R*, *IRF4*, *LYVE1*, *MMP9*, *MMP14*, *MMP19*, *MSR1*, *TGFB1*, *TGFB2*, *TGFB3*, *TNFSF8*, *TNFSF12*, *VEGFA*, *VEGFB*, *VEGFC*). Maturation (*CD40*, *CD80*, *CD86*, *RELB*, *CD83*), regulatory (*CD274*, *PDCD1LG2*, *CD200*, *FAS*, *ALDH1A2*, *SOCS1*, *SOCS2*) and migration (*CCR7*, *MYO1G*, *CXCL16*, *ADAM8*, *ICAM1*, *FSCN1*, *MARCKS*, *MARCKSL1*) signatures were also calculated.

For T-cell signatures, the properTy R package (version 0.0-9; https://cran.r-project.org/web/packages/properties/index.html) was used to calculate and visualize the immune property score, including the cell cycle, exhaustion, cytotoxicity and resident function score, in each subtype.

### Drug sensitivity analysis using scRank

We applied the scRank tool, as described by Li et al. (2024), to infer the sensitivity of different cell types in our scRNA-seq data to immune checkpoint blockades. The scRank tool models drug perturbations by constructing a target-perturbed gene regulatory network and ranks cell types based on their predicted drug response. This approach allowed us to prioritize cell populations for their potential responsiveness to immune checkpoint blockade (ICB) therapy.

### *In vivo* ICB therapy

Orthotopic and subcutaneous tumor-bearing mice were independently and randomly assigned to three treatment groups (*n*=9 per group per model) and received intraperitoneal injections of anti-PDCD1 antibody (clone S-5001, STARTER, 200 μg/dose), anti-CTLA4 antibody (clone 9D9, STARTER, 100 μg/dose) or PBS (100 μl/dose) every three days (six doses in total), starting on day 5 post implantation. Tumor growth was monitored using digital caliper measurements for subcutaneous models and MRI for orthotopic models. Treatment responses were evaluated based on tumor volume dynamics and overall survival. Mice were euthanized upon reaching pre-defined endpoint criteria or humane endpoints.

### Statistical analysis

Statistical analyses were performed using GraphPad Prism version 10. Two-way ANOVA followed by Tukey's multiple comparison test was used to assess statistical differences. Data are presented as error bars showing the ±standard error of the mean (±s.e.m.). Statistical significance was determined at *P*<0.05, with significance levels depicted in figures as *P<0.05, **P<0.01, ***P<0.001 and ****P<0.0001.

Detailed information about the key resources used in this study, including antibodies, cell lines and reagents, is available in the supplementary materials (Table S10 – Key Resources Table).

## Supplementary Material

10.1242/dmm.052311_sup1 Supplementary information

Table S1. Classical Markers of the Five Major Cell Types in Mouse scRNA-seq Samples (Related to Fig. 1)

Table S2. Classical Markers of the Five Major Cell Types in Human scRNA-seq Samples (Related to Fig. 1)

Table S3. Differentially Expressed Genes Across T Cell Clusters in Mouse scRNA-seq (Related to Fig. 2)

Table S4. Differentially Expressed Genes Across T Cell Clusters in Human scRNA-seq (Related to Fig. 2)

Table S5. Differentially Expressed Genes Across Macrophage Clusters in Mouse scRNA-seq (Related to Fig. 3)

Table S6. Differentially Expressed Genes Across Myeloid Clusters in Human scRNA-seq (Related to Fig. 3)

Table S7. Differentially Expressed Genes Across Neutrophil Clusters in Mouse scRNA-seq (Related to Fig. S4)

Table S8. Differentially Expressed Genes Across DC Clusters in Mouse scRNA-seq (Related to Fig. S5)
