# Supplementary material for: Immunological dynamics in orthotopic compared with subcutaneous murine models of HPV-positive oropharyngeal cancer
Source: Dis Model Mech. 2025 Dec 5;18(12):dmm052311. doi: 10.1242/dmm.052311 (PMC12746710; doi:10.1242/dmm.052311)
Supplement: Supplementary information [file dmm-18-052311-s1.pdf]

•

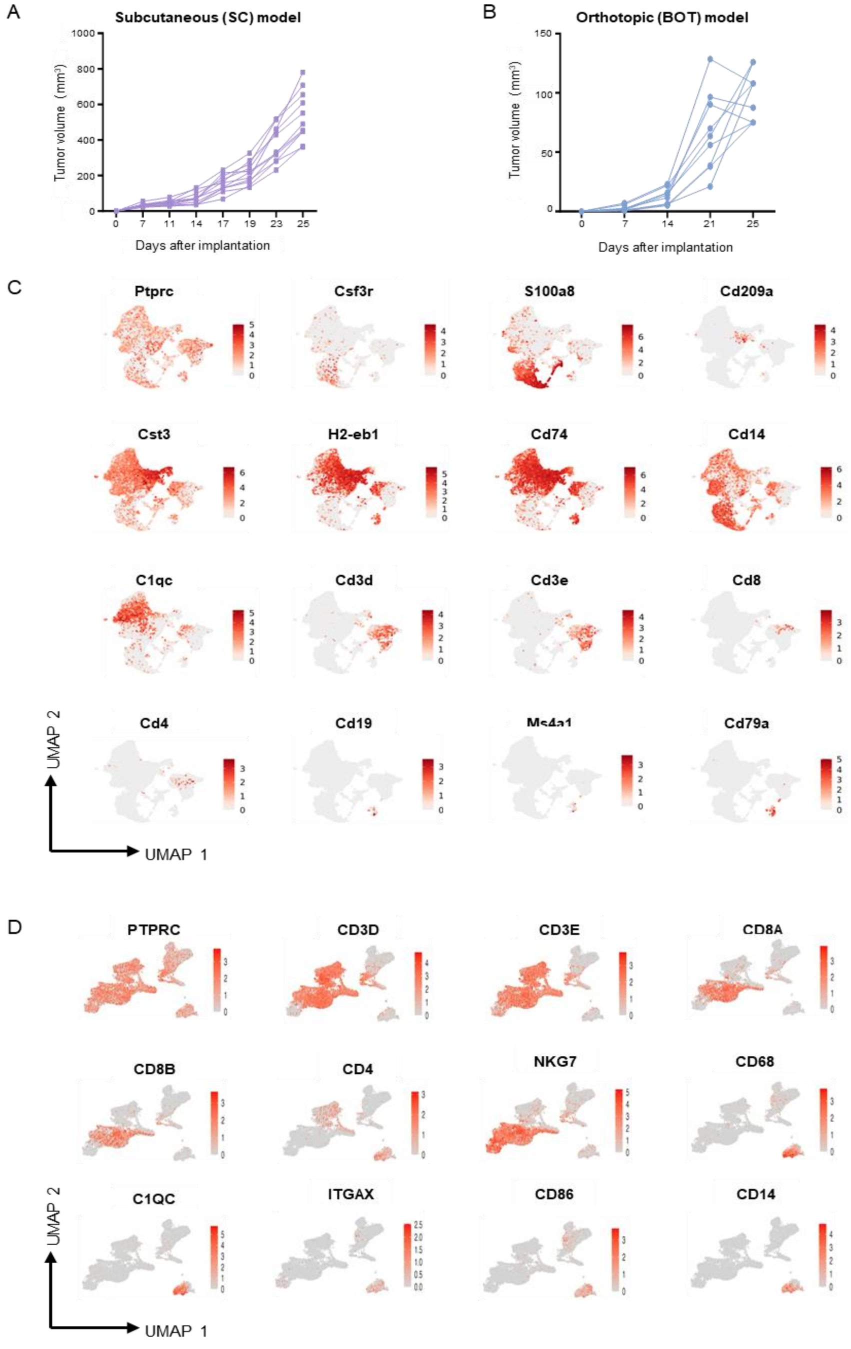

Fig. S1. Tumor growth and immune cell annotation in murine and human HPV⁺ OPSCC.

(A–B) Individual tumor growth curves in SC (A) and BOT (B) murine models (n = 11 and 9 per group, respectively). (C) Canonical immune marker gene expression for cell type annotation in SC and BOT models. (D) Canonical immune marker gene expression for immune cell annotation in human HPV⁺ OPSCC.

Disease Models & Mechanisms • Supplementary information

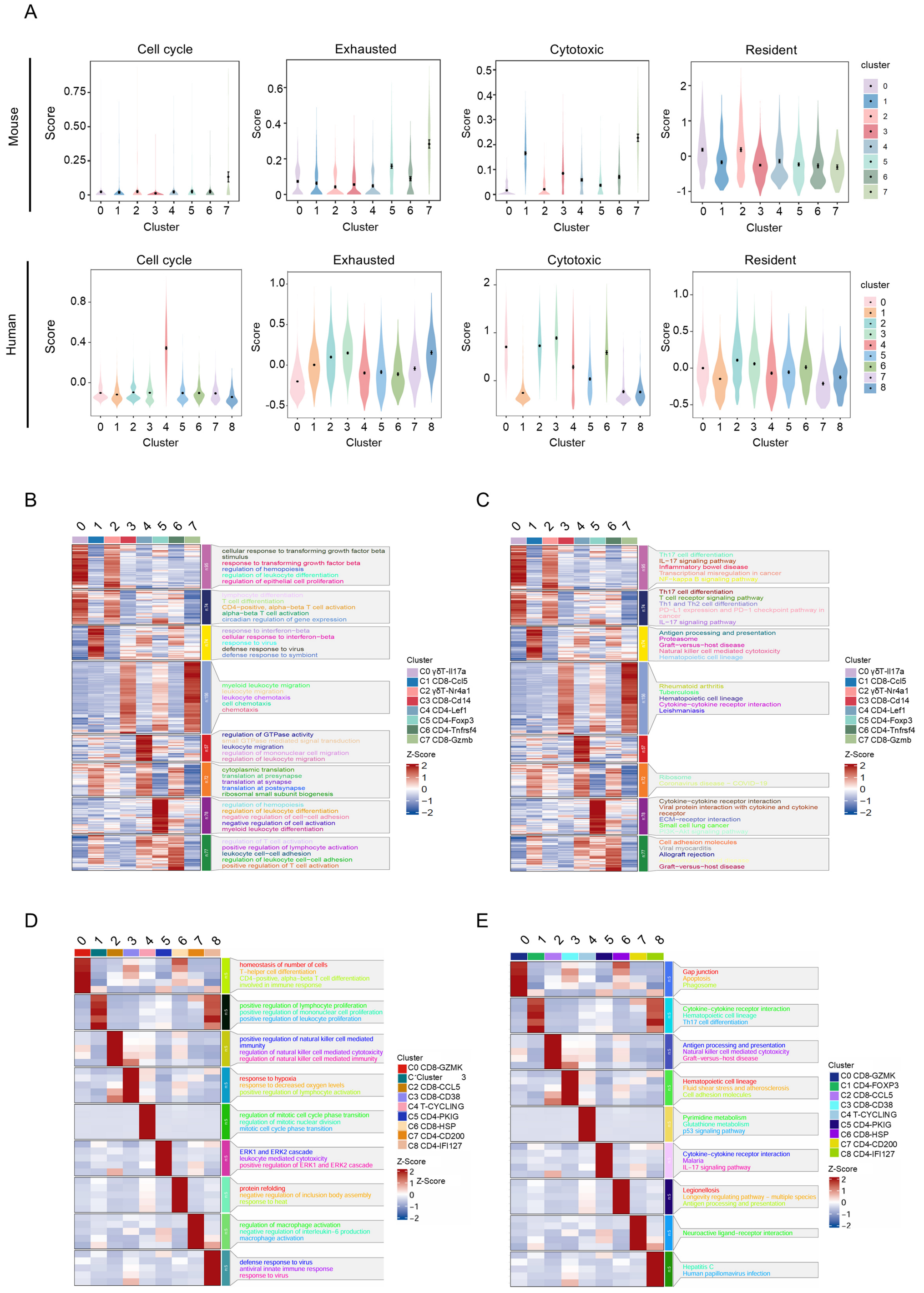

Disease Models & Mechanisms • Supplementary information

Fig. S2. Functional activity and pathway enrichment analysis of T Cell subsets. (A) Violin plots (top) displaying functional scores of T cells in murine models, with corresponding violin plots (bottom) showing functional scores of T cells in human HPV-positive OPSCC. (B-C) Enrichment heatmap of GO (left) and KEGG (right) pathways using specific dropout genes from T cell sub-clusters in murine models. (D-E) Enrichment heatmap of GO (left) and KEGG (right) pathways using specific dropout genes from T cell sub-clusters in human HPV-positive OPSCC.

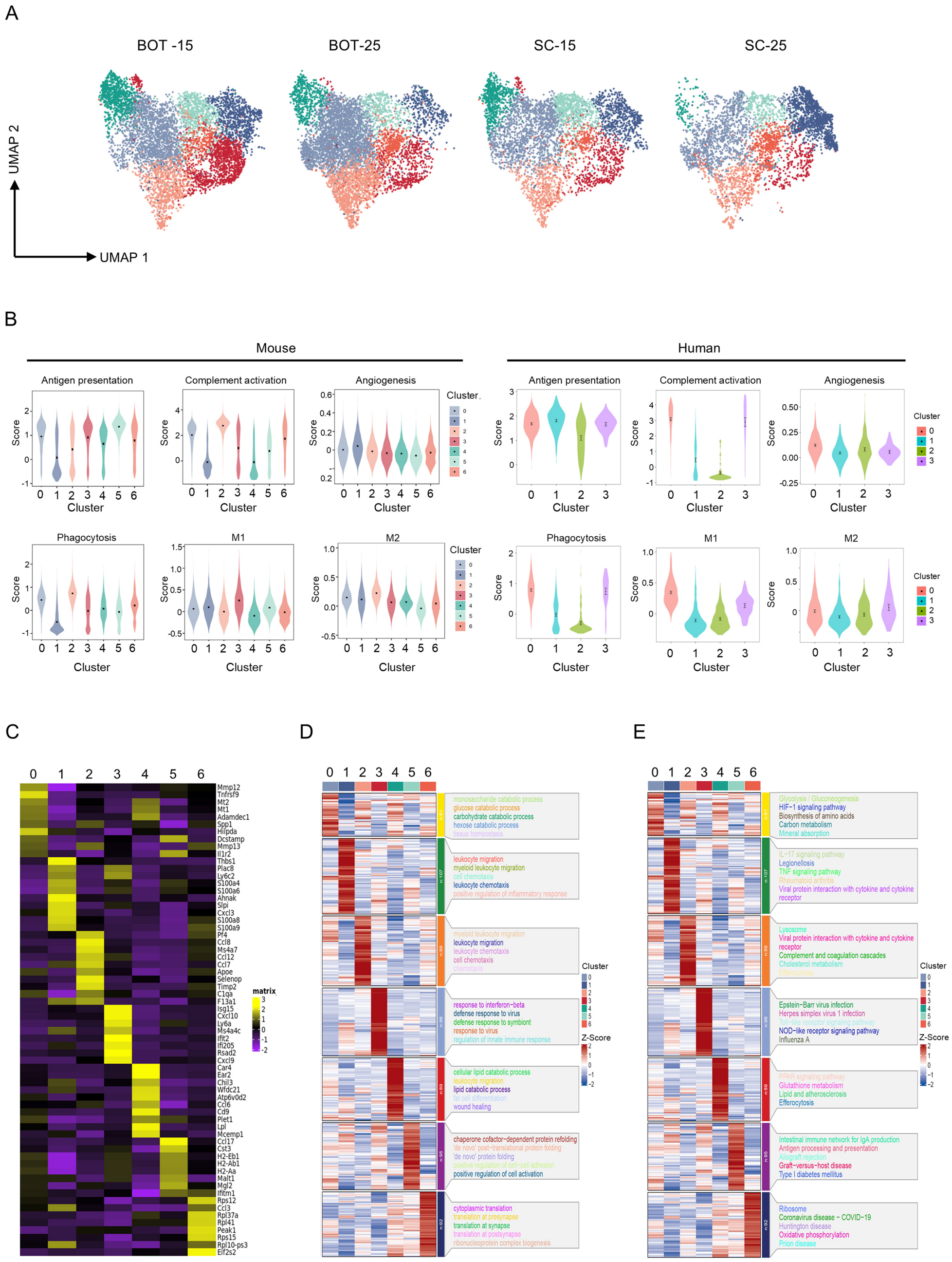
A
BOT -15
BOT-25
SC-15
SC-25
UMAP 2
UMAP 1
B
Mouse
Human
Antigen presentation
Complement activation
Angiogenesis
Phagocytosis
M1
M2
Score
Cluster
C
matrix
D
E
Cluster
Z-Score

Fig. S3. Distribution, transcriptional profiles, and functional characterization of macrophage sub-clusters in murine models. (A) UMAP plots showing the distribution of single macrophage at early (day 15) and late (day 25) stages in BOT and SC murine models. Each dot represents an individual cell, colored according to cell cluster number. (B) Violin plots (left) displaying functional scores of macrophages in murine models, with corresponding violin plots (right) showing functional scores of macrophages in human HPV-positive OPSCC. (C) Heatmap showing the top 10 gene expression across macrophage sub-clusters in murine models. (D) Enrichment heatmap of GO pathways based on specific dropout genes from macrophage sub-clusters in murine models. (E) Enrichment heatmap of KEGG pathways based on specific dropout genes from macrophage sub-clusters in murine models.

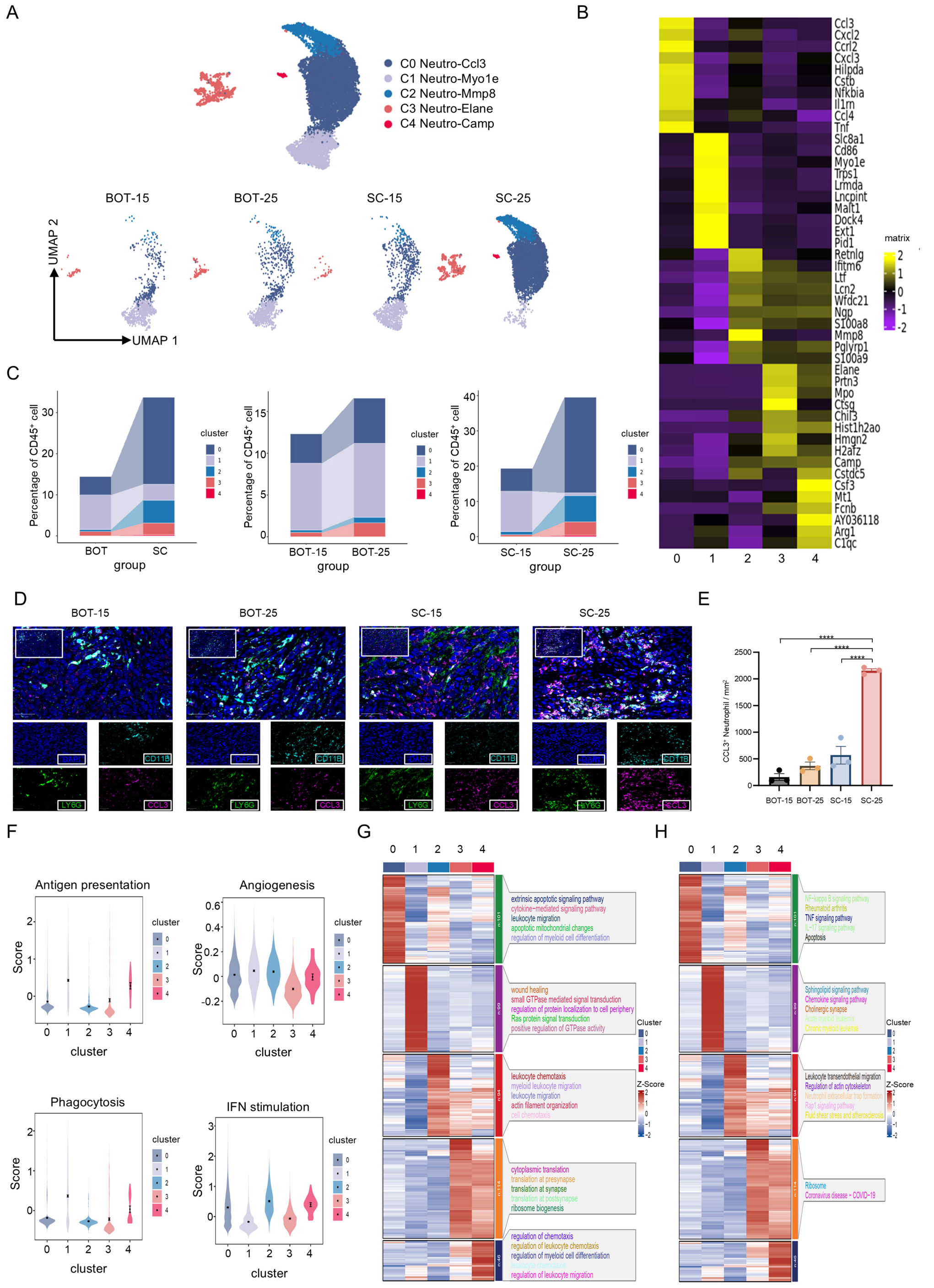

Disease Models & Mechanisms • Supplementary information

Fig. S4. Neutrophil sub-cluster composition and functional pathway enrichment in murine models. (A) UMAP plots (top) of neutrophil showing the formation of 5 sub-clusters. Each dot represents an individual cell, colored according to cell cluster number. UMAP plots (bottom) showing the distribution of single neutrophil at early (day 15) and late (day 25) stages in BOT and SC murine models. (B) Heatmap showing the top 10 gene expression across neutrophil sub-clusters in murine models. (C) Histogram depicting the percentage changes of neutrophil sub-clusters among $CD45^+$ cells between different murine model (BOT vs. SC) and different stage (early vs. late) in the SC and BOT models. (D) Representative immunofluorescence images of SC and or BOT tumors harvested at day 15 and day 25 post-implantation. Sections stained for $CCL3^+$ neutrophils ($CD11B^+$ $LY6G^+$ $CCL3^+$). Scale bar: 50 µm. (E) $CCL3^+$ neutrophils per mm^2 tumor area for each group were presented as mean ± SEM (5 fields/mouse × 3 mice/group). Statistical significance determined by two-way ANOVA with Tukey's post-hoc test: *$P < 0.05$, **$P < 0.01$, ***$P < 0.001$ and ****$P < 0.0001$. (F) Violin plots displaying functional scores of neutrophils in murine models. (G) Enrichment heatmap of GO pathways based on specific dropout genes from neutrophil sub-clusters in murine models. (H) Enrichment heatmap of KEGG pathways based on specific dropout genes from neutrophil sub-clusters in murine models.

Disease Models & Mechanisms • Supplementary information

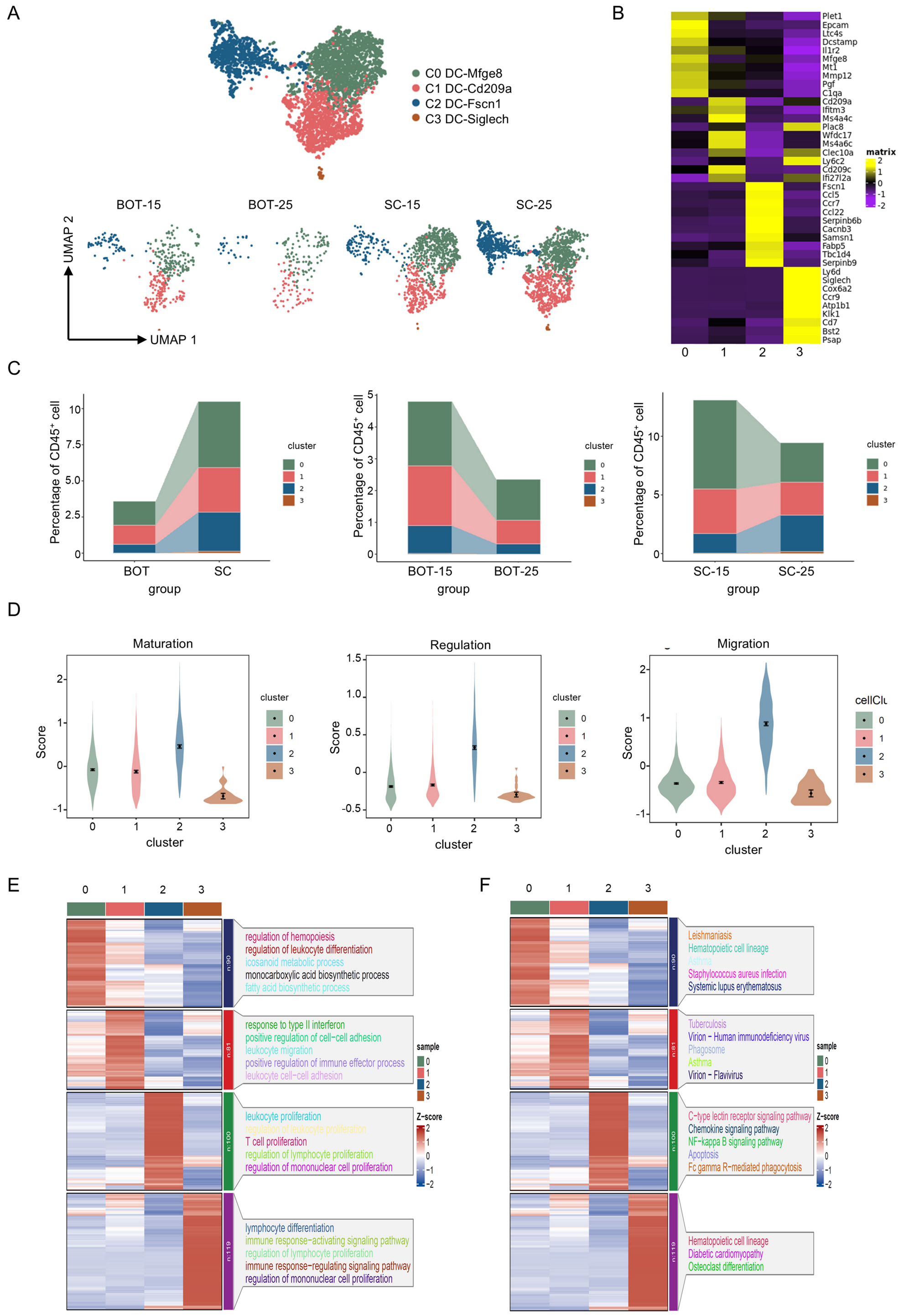

Disease Models & Mechanisms • Supplementary information

Fig. S5. Distribution, Differentially Expressed Genes, Proportion and Phenotype of DC Subclusters in Murine Models. (A) UMAP plots (top) of DC showing the formation of 4 sub-clusters. Each dot represents an individual cell, colored according to cell cluster number. UMAP plots (bottom) showing the distribution of single DC at early (day 15) and late (day 25) stages in BOT and SC murine models. (B) Heatmap showing the top 10 gene expression across DC sub-clusters in murine models. (C) Histogram depicting the percentage changes of DC sub-clusters among CD45^{+} cells between different murine model (BOT vs. SC) and different stage (early vs. late) in the SC and BOT models. (D) Violin plots displaying functional scores of DC in murine models. (E) Enrichment heatmap of GO pathways based on specific dropout genes from DC sub-clusters in murine models. (F) Enrichment heatmap of KEGG pathways based on specific dropout genes from DC sub-clusters in murine models.

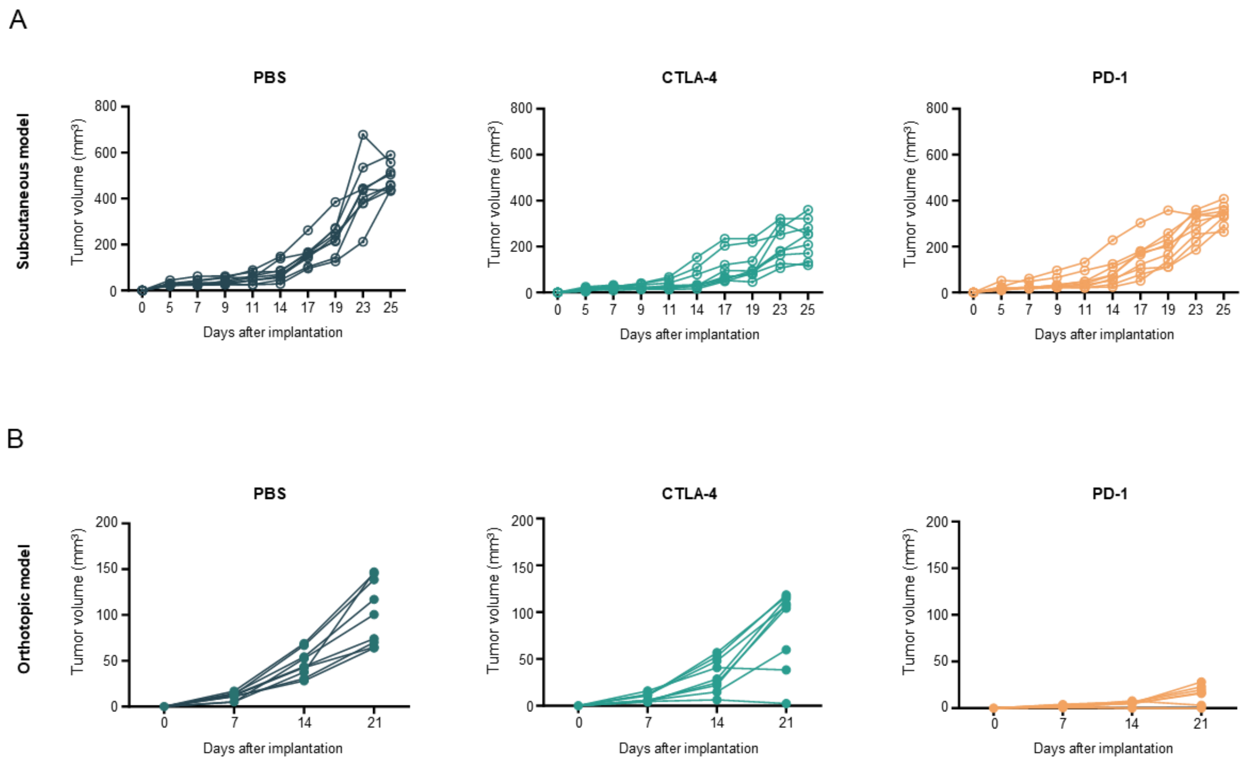

Fig. S6. Individual tumor growth in murine models of HPV⁺ OPSCC under ICB treatment. (A – B) Individual tumor growth curves of each mouse in SC (A) and BOT (B) models treated with PBS, anti-CTLA-4, or anti-PD-1 (n = 9 per group for both models).

Table S1. Classical Markers of the Five Major Cell Types in Mouse scRNA-seq Samples (Related to Fig. 1)

Available for download at
https://journals.biologists.com/dmm/article-lookup/doi/10.1242/dmm.052311#supplementary-data

Table S2. Classical Markers of the Five Major Cell Types in Human scRNA-seq Samples (Related to Fig. 1)

Available for download at
https://journals.biologists.com/dmm/article-lookup/doi/10.1242/dmm.052311#supplementary-data

Table S3. Differentially Expressed Genes Across T Cell Clusters in Mouse scRNA-seq (Related to Fig. 2)

Available for download at
https://journals.biologists.com/dmm/article-lookup/doi/10.1242/dmm.052311#supplementary-data

Table S4. Differentially Expressed Genes Across T Cell Clusters in Human scRNA-seq (Related to Fig. 2)

Available for download at
https://journals.biologists.com/dmm/article-lookup/doi/10.1242/dmm.052311#supplementary-data

Table S5. Differentially Expressed Genes Across Macrophage Clusters in Mouse scRNA-seq (Related to Fig. 3)

Available for download at
https://journals.biologists.com/dmm/article-lookup/doi/10.1242/dmm.052311#supplementary-data

Table S6. Differentially Expressed Genes Across Myeloid Clusters in Human scRNA-seq (Related to Fig. 3)

Available for download at
https://journals.biologists.com/dmm/article-lookup/doi/10.1242/dmm.052311#supplementary-data

Table S7. Differentially Expressed Genes Across Neutrophil Clusters in Mouse scRNA-seq (Related to Fig. S4)

Available for download at
https://journals.biologists.com/dmm/article-lookup/doi/10.1242/dmm.052311#supplementary-data

Table S8. Differentially Expressed Genes Across DC Clusters in Mouse scRNA-seq (Related to Fig. S5)

Available for download at
https://journals.biologists.com/dmm/article-lookup/doi/10.1242/dmm.052311#supplementary-data

Table S9. Metadata of Public Datasets Included in This Study (Related to Fig. 1)

Stage	PubmedID	Data accession number	PatientID	SampleID	Clinical stages
T1	37012457	GSE182227	OPSCC20	GSM5525405	Early
T1	37012457	GSE182227	OPSCC33	GSM5525406	Early
T1	36828832	GSE181919	P57	GSM5514363	Early
T1	38857913	GSE226620	HPV02	GSM7080428	Early
T1	38857913	GSE226620	HPV03	GSM7080429	Early
T1	38857913	GSE226620	HPV08	GSM7080434	Early
T2	37012457	GSE182227	OPSCC17	GSM5525403	Early
T2	37012457	GSE182227	OPSCC34	GSM5525408	Early
T2	36828832	GSE181919	P22	GSM5514355	Early
T2	36828832	GSE181919	P46	GSM5514361	Early
T2	36828832	GSE181919	P84	GSM5514368	Early
T2	36828832	GSE181919	P86	GSM5514369	Early
T2	38857913	GSE226620	HPV01	GSM7080427	Early
T2	38857913	GSE226620	HPV04	GSM7080430	Early
T2	38857913	GSE226620	HPV05	GSM7080431	Early
T3	37012457	GSE182227	OPSCC5	GSM5525391	Late
T3	36828832	GSE181919	P43	GSM5514360	Late
T3	36828832	GSE181919	P59	GSM5514364	Late

Table S10. Key Resources table.

REAGENT or RESOURE	SOURCE	IDENTIFIER	RRID
**Antibodies**			
7-AAD viability staining solution	BioLegend	Cat# 420404	AB_2563359
Anti-mouse CD45 (clone 30-F11)	BioLegend	Cat# 103130	AB_312979
CD45 (D3F8Q) Rabbit mAb	CST	Cat# 70257	AB_2799781
CD3ε (E4T1B) XP® Rabbit mAb	CST	Cat# 78588	AB_2728761
Anti-CD8 alpha antibody [EPR21769]	Abcam	Cat# ab217344	AB_2890649
Granzyme B (E5V2L) Rabbit mAb	CST	Cat# 44153	AB_2799263
CD4 (D7D2Z) Rabbit mAb	CST	Cat# 25229	AB_2798897
FoxP3 (D6O8R) Rabbit mAb	CST	Cat# 12653	AB_2797979
Anti-CD19 antibody [EPR23174-145]	Abcam	Cat# ab245235	AB_2890648
Anti-CD11b antibody [EPR1344]	Abcam	Cat# ab133357	AB_2650514
CD11c (D1V9Y) Rabbit mAb	CST	Cat# 97585	AB_2800250
F4/80 (D4C8V) XP® Rabbit mAb	CST	Cat# 30325	AB_2798960
Ly-6G (E6Z1T) Rabbit mAb	CST	Cat# 87048	AB_2728764
Anti-Mouse TCR Gamma/Delta Antibody (clone GL3)	STEMCELL	Cat# 60104AD.1	AB 3698197
IL-17a Polyclonal antibody	Proteintech	Cat# 26163-1-AP	AB_2880947
Osteopontin/SPP1 (E9Z1D) Rabbit mAb	CST	Cat# 88742	AB_2800093
CCL3/MIP-1alpha (F2M8Q) Rabbit mAb	CST	Cat# 85270	AB_2800087
In vivo anti-mouse CTLA-4 mAb (clone 9D9)	STARTER	Cat# S0B0574	N/A
In vivo anti-mouse PD-1 Recombinant mAb (clone S-5001)	STARTER	Cat# S0B0594	N/A

**Critical commercial assays**			
AO/PI double staining kit	absin	Cat# abs9727-100T	N/A
RPMI 1640 culture medium	Gibco	Cat# C11875500BT	N/A
Phosphate Buffered Saline (PBS)	Biosharp	Cat# BL302A	N/A
collagenase type II	Gibco	Cat# 17104019	N/A
collagenase type IV	Gibco	Cat# 17104015	N/A
Deoxyribonuclease I	Sigma Aldrich	Cat# 11284932001	N/A
Bovine Serum Albumin (BSA)	Biofroxx	Cat# 4240GR025	N/A

Pancreatic enzymes TRYPSIN 0.25% EDTA	Gibco	Cat# 25200056	N/A
Penicillin-Streptomycin	Hyclone	Cat# SV30010	N/A
Fetal Bovine Serum (FBS)	Procell	Cat#164210	N/A
Prigrow IV culture medium	abm	Cat# TM004	N/A
**Experimental models: Cell lines**			
mEERL HPV^{+} oropharyngeal cancer cell line	Applied Biological Materials (abm)	Cat# T8309 (RRID: CVCL_B6J3)	CVCL_B6J3
**Experimental models: Organisms/strains**			
Mouse: wild-type C57BL/6J mice	SPF (Beijing) Biotechnology Co., Ltd.	C57BL/6JNCrl	IMSR_JAX:000664
**Software and algorithms**			
3D Slicer (v5.6.2)	3D Slicer	https://www.slicer.org/	SCR_005619
Qupath v0.5.1	University of Edinburgh	https://qupath.github.io/	SCR_018257
Prism (v10)	GraphPad	https://www.graphpad.com/	SCR_002798
Cell Ranger (v3.1.0)	10x Genomics	http://10xgenomics.com/	SCR_017344
Seurat (v4.0)	Hao et al. 2021	https://satijalab.org/seurat/	SCR_016341
harmony (v0.1.0)	Korsunsky et al. 2019	https://github.com/immunogenomics/harmony	SCR_018919
slingshot (v2.2.1)	Street, K et al. 2018	https://www.bioconductor.org/packages/release/bioc/html/slingshot.html	SCR_017012
SingleCellExperiment (v1.16.0)	Amezquita R et al. 2020	https://bioconductor.org/packages/release/bioc/html/SingleCellExperiment.html	SCR_027818
pheatmap (v1.0.12)	CRAN	https://rdrr.io/cran/pheatmap/	N/A

Disease Models & Mechanisms • Supplementary information

edgebundleR (v0.1.5)	NA	https://github.com/garthtarr/edgebundleR	N/A
scRank (v1.0.0)	Chengyu Li et al. 2024	https://github.com/ZJUFanLab/scRank	SCR_028683
clusterProfiler (v4.10.0)	Tianzhi Wu et al. 2021	https://github.com/GuangchuangYu/enrichment4GTEx_clusterProfiler	N/A

Disease Models & Mechanisms • Supplementary information
